# Incompatible *Aedes aegypti* male releases as an intervention to reduce mosquito population—A field trial in Puerto Rico

**DOI:** 10.1371/journal.pntd.0012839

**Published:** 2025-01-21

**Authors:** Liliana Sánchez-González, Jacob E. Crawford, Laura E. Adams, Grayson Brown, Kyle R. Ryff, Mark Delorey, Jose Ruiz-Valcarcel, Nicole Nazario, Nexilianne Borrero, Julieanne Miranda, Sara N. Mitchell, Paul I. Howell, Johanna R. Ohm, Charlie Behling, Brian Wasson, Craig Eldershaw, Bradley J. White, Vanessa Rivera-Amill, Roberto Barrera, Gabriela Paz-Bailey

**Affiliations:** 1 Division of Vector Borne Diseases, Centers for Disease Control and Prevention, San Juan, Puerto Rico; 2 Verily Life Sciences, San Francisco, California, United States of America; 3 Puerto Rico Vector Control Unit, San Juan, Puerto Rico; 4 Ponce Health Sciences University/Ponce Research Institute, Ponce, Puerto Rico; University of California Davis School of Veterinary Medicine, UNITED STATES OF AMERICA

## Abstract

Mosquito-transmitted viruses such as dengue are a global and growing public health challenge. Without widely available vaccines, mosquito control is the primary tool for fighting the spread of these viruses. New mosquito control technologies are needed to complement existing methods, given current challenges with scalability, acceptability, and effectiveness. A field trial was conducted in collaboration with the Communities Organized to Prevent Arboviruses project in Ponce, Puerto Rico, to measure entomological and epidemiological effects of reducing *Aedes aegypti* populations using *Wolbachia* incompatible insect technique. We packed and shipped *Wolbachia*-males from California and released them into 19 treatment clusters from September 2020 to December 2020. Preliminary evaluation revealed sub-optimal *Wolbachia*-male densities and impact on the wild-type population. In 2021, we shifted to a phased release strategy starting in four clusters, reducing the mosquito population by 49% (CI 29–63%). We describe the investigation into male quality and other factors that may have limited the impact of *Wolbachia*-male releases. Laboratory assays showed a small but significant impact of packing and shipping on male fitness. However, mark-release-recapture assessments suggest that male daily survival rates in the field may have been significantly impacted. We compared induced-sterility levels and suppression of the wild population and found patterns consistent with mosquito population compensation in response to our intervention. Analysis of epidemiological impact was not possible due to very low viral transmission rates during the intervention period. Our entomological impact data provide evidence that *Wolbachia* incompatible-male releases reduced *Ae*. *aegypti* populations, although efficacy will be maximized when releases are part of an integrated control program. With improvement of shipping vessels and shipped male fitness, packing and shipping male mosquitoes could provide a key solution for expanding access to this technology. Our project underscores the challenges involved in large and complex field effectiveness assessments of novel vector control methods.

## Author summary

Mosquito control is a key component of strategies to reduce the increasing occurrence of mosquito-borne diseases like dengue. New effective technologies are needed to complement existing methods. In the *Wolbachia*-based incompatible insect technique, male mosquitoes with *Wolbachia* are released repeatedly to mate with wild female mosquitoes; the resulting eggs don’t hatch, hence reducing the mosquito population over time. We assessed the effectiveness of this technique using mosquitoes reared and packed in California and shipped and released in 19 community clusters in Ponce, Puerto Rico. Analysis of the impact of the releases in decreasing dengue occurrence was not possible, given the low transmission rate during the project period. Preliminary analysis showed sub-optimal mosquito population reduction; we then focused the releases in 4 clusters, reaching up to 49% (CI 29–63%) population reduction. Our extensive laboratory investigations suggest that the packing and shipping process had significant effects on mosquito fitness and daily survival rates, which may have limited the impact of the releases and prevented reaching a higher population reduction. Our data confirms that this technique is effective in reducing mosquito populations in Ponce, PR, but efficacy can increase when releases are part of an integrated control strategy.

## Introduction

Arboviruses transmitted by *Aedes* mosquitoes, including dengue, Zika, and chikungunya viruses, are a growing public health threat in tropical and subtropical regions, with the frequency and magnitude of their outbreaks increasing globally in recent decades [1,2]. This increase has been attributed to a combination of factors, including human movement, poorly planned urbanization, and the expansion of suitable climate for the vectors, *Aedes aegypti* and *Aedes albopictus*, that have increased their geographic distribution [3–5].

In Puerto Rico, where dengue is endemic, outbreaks usually occur every 3 to 5 years [6]. More than 18,000 suspected cases, 9,200 confirmed cases, and 12 deaths were reported during the last epidemic in 2012–2013 [7], and in 2022, more than 1,000 confirmed and probable dengue cases were reported on the island [8]. In addition to dengue, a chikungunya outbreak occurred in Puerto Rico in 2014 with more than 4,500 confirmed cases, followed by a Zika outbreak in 2016 with more than 36,000 confirmed cases [9–12]

Vector control continues to be the primary prevention approach available for these arboviruses [13], but commonly used methods face important challenges including scalability, acceptability, and effectiveness. For example, widespread resistance to pyrethroids has been documented among *Ae*. *aegypti* in Puerto Rico, limiting the effectiveness of insecticides used for widespread vector control on the island [14,15]. Therefore, assessing the effectiveness and implementation challenges of existing and new vector control interventions continues to be crucial in the fight against arboviral diseases.

Mass releases of sterile male insects (Sterile Insect Technique, SIT) have been used as vector control methods for different insect species [16–19]. Male mosquitoes infected with *Wolbachia pipientis*, a gram-negative endosymbiotic bacterium, can be reproductively incompatible with females that do not carry the same *Wolbachia* strain, resulting in eggs that do not hatch [20]. This method, now called Incompatible Insect Technique (IIT) has been used previously in multiple locations including several states in the United States [21–23], Singapore [24], French Polynesia [25], and Australia [26] with demonstrated population reductions of up to 99% (78% - 99%). Effective suppression using IIT or SIT depends on frequent releases to maintain consistently high densities of fit, released males in the landscape to outcompete wild males. SIT and IIT can be used in combination with other vector control methods, including source control and larvicide applications, in an integrated manner [27].

Communities Organized to Prevent Arboviruses (COPA) is a community-based prospective cohort established in Ponce, Puerto Rico in 2018 through a collaboration between the Ponce Health Sciences University, the Puerto Rico Vector Control Unit (PRVCU) and the Dengue Branch of the Centers for Disease Control and Prevention [28–30]. The COPA project works in 38 distinct clusters selected across the Ponce municipality and aims to assess the risk of infection with dengue and other arboviruses and serve as a platform to evaluate the acceptability and effectiveness of vector control and other prevention methods. Formative research and COPA’s participant surveys showed high acceptability of the *Wolbachia* IIT. During the first year of the cohort in 2018, 67% (1,037/1,528) of participants surveyed supported *Wolbachia* suppression. A follow-up survey in 2020, during and after an educational campaign, showed that most COPA participants (86%; 333/389)) and other COPA communities’ residents (84%; 2,328/2,756) supported the releases of male mosquitoes with *Wolbachia* [29]. In this project, we aimed to determine the effectiveness of the release of male mosquitoes with *Wolbachia* (wAlbB) to reduce *Ae*. *aegypti* mosquito population densities to decrease dengue transmission in COPA communities in Ponce, Puerto Rico. Here, we describe two phases of the suppression intervention. In Phase I, we released *Wolbachia* male mosquitos in half (n = 19) of the COPA clusters, randomly assigned as treatment clusters. Early monitoring data showed that the wild population of *Ae*. *aegypti* was not sufficiently reduced, so we adjusted the release strategy in Phase II to concentrate releases in four of the 19 treatment clusters with the goal of suppressing all treatment clusters over time. Given dengue transmission among COPA participants was very low during the project period (only one participant with a positive dengue antibody test result), reflecting the low transmission on the island, and suppression levels were only moderate, Phase II releases were terminated before expanding to all 19 treatment clusters. We report on entomological outcomes from both phases.

## Methods

### Ethics statement

The COPA study was reviewed and approved by Ponce Medical School Foundation, Inc. Institutional Review Board (protocol number 171110-VR).

### Overview of project phases

The project included two major phases. After pre-release activities including mosquito population monitoring and community outreach, Phase I began in September of 2020. The overall goal of the project was to test whether suppressing the wild population of *Ae*. *aegypti* in 19 communities of Ponce, Puerto Rico, would lead to a statistically significant reduction in dengue case rates in people who live in these communities compared to 19 other communities where no mosquito control was undertaken. In Phase I, we released *Ae*. *aegypti Wolbachia* male mosquitoes into all 19 treatment clusters with the goal of suppressing all 19 treatment clusters at the same time. In December of 2020, mosquito monitoring data showed that the wild population was not sufficiently reduced after three months of releases. In response, we adjusted the release strategy in Phase II (January 2021) and focused releases in four clusters to start, resulting in substantially increased densities of released males relative to wild *Ae*. *aegypti* male densities. We continued releases in the first four clusters until the end of the project in December of 2021. As in Phase I, the goal of Phase II was to suppress the wild population of *Ae*. *aegypti* in all 19 treatment clusters. However, after one year of releases in Phase II with only moderate impact on the wild mosquito population and insufficient dengue cases for statistical measurement, the project was ended in December 2021.

### COPA setting and methods

**Fig 1:**
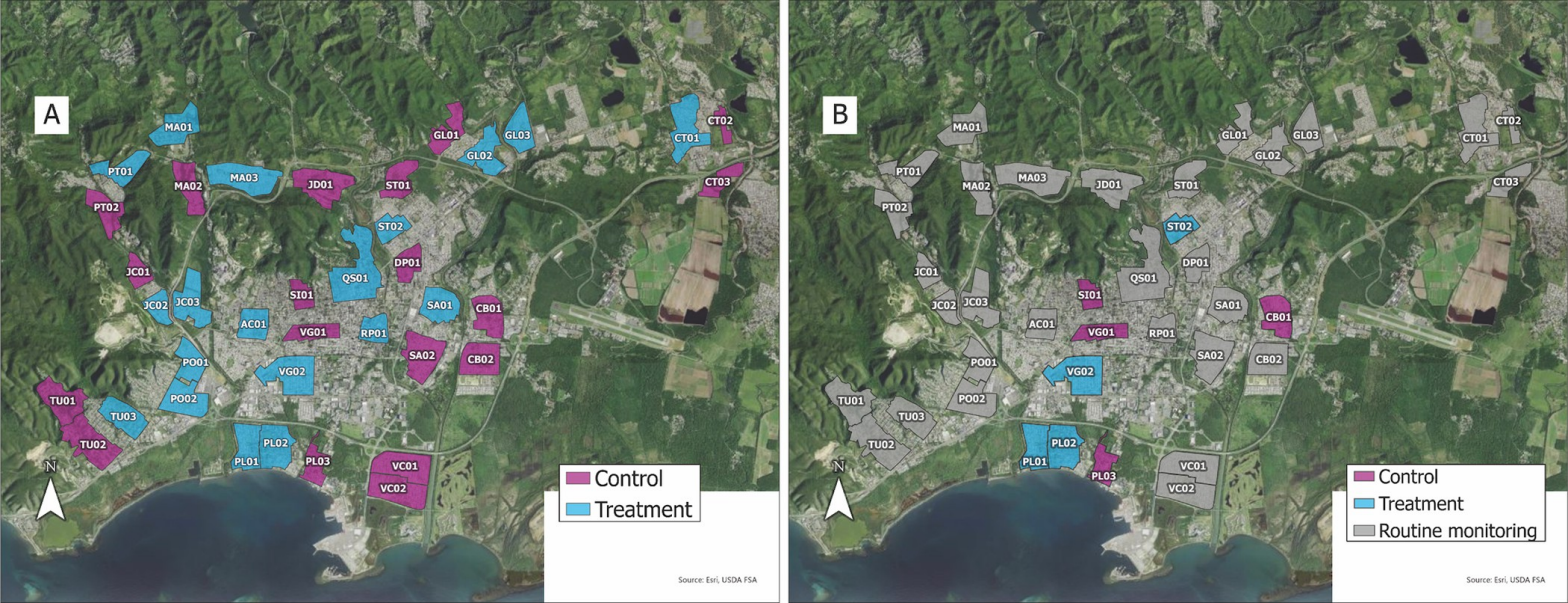
Location and project assignment of COPA clusters, Ponce, Puerto Rico, September 2020–December 2021. A) 38 polygons showing clusters defined for the COPA study. Phase I releases were conducted in 19 treatment clusters (blue) in 2020. B) Phase II treatment clusters are highlighted in purple and control clusters highlighted in blue. Remaining clusters received no extra activity, but monitoring traps remained active during Phase II. Map source: USDA Farm Service Agency, National Agriculture Imagery Program. Map created using ArcGIS Pro 2.6.0. USDA:NRCS:Geospatial Data Gateway:Home:Direct DownLoad.

The COPA project was initiated in 2018, and categorized areas of Ponce, Puerto Rico into distinct study clusters organized by previous arboviral incidence data in Ponce, social and geographic neighborhood delimitation and size, engagement with community leaders, safety issues, and type of housing [31]. The implementation and completion of the *Wolbachia* male mosquito releases were preceded by a broad educational campaign that continued during the releases. Public perception of the COPA study and the releases were very positive during and after the project period, with very little opposition voiced. To minimize “contamination” (mosquitoes flying from treatment clusters to control clusters) the 38 clusters were separated from each other by a distance of at least 150 meters, the typical flight distance of *Ae*. *aegypti* males [32] or by a geographical barrier such as a river, forest, or major roadway, except when adjacent clusters were assigned the same treatment or control status (Fig 1). The clusters ranged in size from 15 to 126 hectares (ha) (Table 1) and spanned the city from the coast to the hills with a mixture of residential and non-residential properties (Fig 1). COPA study clusters and methods have been described in previous publications [28–30]. Briefly, ~3,800 COPA participants ages 1–50 years, residing in 38 distinct clusters have annual interviews including standardized questionnaires on socio-demographics, health status, history of febrile illness, and other health-related questions, as well as providing blood samples for arboviral testing. Through text messaging, participants are also asked every week about fever and other symptoms and offered arboviral and respiratory viruses testing. The COPA study design was powered to detect a 50% reduction in arboviral disease incidence among 3,800 participants over a three-year intervention period [30].

**Table 1:** Characteristics of COPA clusters (N = 38), Ponce, Puerto Rico, 2020–2021.

Cluster	Category	Area (ha)	Estimated number of structures	Number of mosquito surveillance traps	Traps per ha
AC01	Treatment	55.06	1220	15	0.27
CT01	Treatment	82.58	1614	16	0.19
GL02	Treatment	52.28	806	14	0.27
GL03	Treatment	38.93	706	16	0.41
JC02	Treatment	28.31	580	17	0.6
JC03	Treatment	50.07	973	16	0.32
MA01	Treatment	42.35	495	19	0.45
MA03	Treatment	70.15	1138	20	0.29
PL01	Treatment	43.91	609	23	0.52
PL02	Treatment	59.53	825	19	0.32
PO01	Treatment	46	922	15	0.33
PO02	Treatment	60.34	1293	13	0.22
PT01	Treatment	42.68	823	15	0.35
QS01	Treatment	125.84	3237	25	0.2
RP01	Treatment	51.65	1209	15	0.29
SA01	Treatment	62.11	867	15	0.24
ST02	Treatment	54.28	760	19	0.35
TU03	Treatment	67.22	960	17	0.25
VG02	Treatment	91.7	1629	25	0.27
**Total**	**Treatment**	**1124.99**	**20666**	334	0.30
CB01	Control	43.73	682	18	0.41
CB02	Control	49.07	868	15	0.31
CT02	Control	15.13	282	13	0.86
CT03	Control	30.01	109	14	0.47
DP01	Control	30.37	265	12	0.4
GL01	Control	39.61	613	14	0.35
JC01	Control	23.8	478	15	0.63
JD01	Control	62.72	1140	16	0.26
MA02	Control	42.64	591	15	0.35
PL03	Control	29.47	681	13	0.44
PT02	Control	39.92	497	13	0.33
SA02	Control	59.1	1008	13	0.22
SI01	Control	22.47	834	13	0.58
ST01	Control	39.28	816	15	0.38
TU01	Control	54.75	786	15	0.27
TU02	Control	67.43	902	14	0.21
VC01	Control	58.23	1219	15	0.26
VC02	Control	59.7	863	14	0.23
VG01	Control	31.75	594	15	0.47
**Total**	**Control**	**799.17**	**13228**	272	0.34

### Approvals/Permits

The open release of male *Ae*. *aegypti* mosquitoes with *Wolbachia* was approved in 2016 under an Experimental Use Permit (EUP; 89668-EUP-3 2020-12-04) by the United States Environmental Protection Agency (EPA), which expired at the end of 2021 [33]. In 2020, the Puerto Rico Department of Agriculture issued the required permits for the PRVCU to release male mosquitoes with *Wolbachia* in Ponce. Commercial use of the WB1 isolate of *Wolbachia* in *Aedes aegypti* male mosquitoes was granted in the United States on April 16, 2024 by the EPA [34].

### Cluster assignment to treatment and control

Based on previously available prevalence data for CHIKV infection, clusters were assigned to one of three categories: 1 = low prevalence (< 28%), 2 = medium prevalence (>28–43%), 3 = high prevalence (>44%). Randomization was then conducted for clusters in each category using the sample R function, which is based on a random number generator without replacement [35]. The location of the 38 clusters and their classification as treatment or control clusters is shown in Fig 1A and cluster characteristics are described in Table 1.

In Phase I of the project from September 3, 2020 to December 29, 2020, *Wolbachia* males were released into all 19 treatment clusters. In January 2021, to increase suppression levels, Phase II releases were concentrated into four clusters as part of a phased release strategy similar to a rolling carpet or stepped-wedge design [27], but organized into isolated clusters rather than contiguous release areas. We started with four clusters based on preliminary data suggesting that splitting shipments among the number of households in four clusters would lead to male densities necessary to reach the targeted overflooding ratio of 10 released males to 1 wild male, based on successful suppression in previous studies with ratios ranging from 6 to 18 released males for every one wild male [21,26].

For Phase II releases, we ranked the 19 treatment clusters based on disease transmission risk, which we calculated using historical *Ae*. *aegypti* female densities as well as an estimate of the proportion of residents under the age of 20 that are, therefore, likely to be susceptible to dengue infection. We selected the top ranked four treatment clusters that could be treated at one time within logistical and release capacity constraints to improve the probability of detecting an epidemiological effect under this phased strategy. This selection process led us to select PL01, PL02, ST02, and VG02 (Fig 1) based on their combined size as well as their high transmission risk. We chose four control clusters that approximately matched the four treatment clusters in size and pre-release female densities such that the starting pre-release suppression level was approximately zero.

### Monitoring

Adult *Ae*. *aegypti* mosquitoes were monitored using Autocidal Gravid Ovitraps (AGOs) [36,37] deployed in treatment and control clusters. Trap deployment began in some clusters in April 2018 and were finalized in September 2020 before the start of *Wolbachia* male releases. Trap densities are listed in Table 1, with an average of one trap per 3.2 ha. Traps were visited every week to obtain adult mosquito counts, and full replacement of water, hay, and glue paper was conducted every eight weeks according to standard protocols for AGOs. Monitoring with AGO traps continued until traps were removed during the last week of March 2022, three months after the completion of the releases. To maximize limited resources, AGOs were the primary measure of impact in Phase I. Ovitraps and other traps were not used in Phase I.

We confirmed the presence of an established *Ae*. *aegypti* population in all clusters with a range of adult mosquito densities. Baseline counts before *Wolbachia* mosquito releases are shown in S1 Fig. We hypothesized that we could achieve sufficiently high overflooding ratios to have an impact on the population based on approximate estimates of the wild population stemming from the mean number of females per trap night in the treatment clusters.

In Phase II, additional monitoring was needed to increase male mosquito capture rates in order to measure the ratio of W*olbachia* males to wild males. We added 15 BG Sentinel v2 traps (Biogents, Regensburg, Germany) in March 2021 to each of the four Phase II treatment clusters (average of one BG per 3.3 ha). Traps were deployed with BG lures and powered by 12v 35Ah batteries. Traps were active for three trap nights per collection and collected one time per week. Trap fan speed was recorded using a Kestrel 2000 (Nielsen-Kellerman Company, Boothwyn, PA, USA). Collections with a fan speed less than 4 mph were excluded from analysis. Collected male mosquitoes were shipped to Verily in South San Francisco, tested for *Wolbachia* DNA using a Loop-mediated isothermal amplification (LAMP) assay, and analyzed to calculate overflooding ratio as described previously [21]. In addition to AGO and BG traps, we added ovitraps in Phase II in order to obtain better measurements of *Wolbachia* male mating rates in the release sites. We deployed 30 ovitraps in each of the four treatment and four control clusters (average of one trap per 1.6 ha) to collect eggs for hatch rate assessment as described previously [21]. Briefly, ovitraps were standard black cups with seed germination paper (38# Anchor Paper Inc., St. Paul, MN, USA) and fermented hay infusion [21]. Traps were deployed as pairs with traps on opposite sides of a property for 3 trap nights. Positive egg papers were stored in an insectary (28°C, ~70–80% relative humidity) for >72hrs [38], flooded using a solution of bovine liver powder and yeast, and hatched larvae counted. Induced sterility was calculated using Abbott’s formula [39] and confidence intervals were calculated using 10,000 bootstrap samples.

To confirm that a population of *Ae*. *aegypti* with wAlbB *Wolbachia* had not established in the treatment areas via accidental release of females with *Wolbachia*, we sampled larvae from ovitraps in the treatment clusters and test for *Wolbachia* using the LAMP assay as described above. We tested a total of 135 collections spanning four months in 2021 in all four Phase II treatment clusters. No larvae were positive for *Wolbachia*.

### Mosquito rearing and shipping

The PRVCU partnered with Verily Life Sciences for the production and shipment of male *Ae*. *aegypti* mosquitoes with *Wolbachia*. All *Wolbachia* mosquitoes used in this project carried the WB1 isolate of wAlbB [40]. We collected eggs from multiple locations in Ponce, Puerto Rico and created a wild-type colony. Males from this colony were backcrossed with WB1 females for five generations to generate a backcrossed WB1 colony with Puerto Rican background genetics. We sequenced samples of genomes from the parental colonies and the backcross colony and confirmed the background genetics matched the Puerto Rican parental colony at a rate of 95% on average. Mosquitoes were mass reared, and sex separated at Verily facilities in San Francisco, as described previously [21].

Males were packed and shipped up to five times per week. Several versions of the shipping containers were developed and modified during the project period, but the following is a description of the version used throughout all phases of the project. After sex-sorting, males were chilled and placed into small containers using proprietary processes and materials. Small containers were packed into vessels that were packed into larger custom boxes to maintain cool temperature during transit from San Francisco to the PRVCU lab facility in Ponce.

Based on preliminary laboratory testing, we determined that male mosquitoes would be most effective in the field if they could be delivered to the field site in Ponce and unpacked within 24 hours of sedation and packing. After arrival in Puerto Rico, mosquito containers were required to undergo an inspection by the Puerto Rico Department of Agriculture that could be delayed depending on the time and day of arrival. Once cleared, the containers were transported by van for approximately 1.5 hours to Ponce, Puerto Rico, where the mosquitoes were unpacked at the PRVCU laboratory into release tubes with 10% sugar water as described previously [21] to then be released in the selected treatment communities according to a predetermined release schedule. A summary of the transportation logistics from San Francisco to Ponce is presented in S2 Fig.

### Mosquito releases

A modified van-based delivery system developed by Verily was used for the male releases, as described previously [21]. The delivery system uses a computer-controlled Google maps-based automated system to deliver the male mosquitoes at select release points along a release map. A predetermined number of males were puffed onto the road edge via holes in the passenger side of the vehicle as the vehicle was moving.

We calibrated the number of males to be released in each of the treatment clusters to be proportionate to the approximate number of households in each cluster. Each shipment was divided among treatment clusters to maintain correct proportions within each week. Van-based releases were adjusted at various points during the project in response to consistently high female collections in some of the AGO traps. Adjustments were made to increase the rate of release from the van on given streets in order to increase the density in certain areas of the cluster with higher female numbers. In Phase I, mosquitoes were released three times per week in each treatment cluster following a rotating schedule to cover the 19 clusters, with releases occurring Monday through Saturday. Preliminary analyses of this phase results showed that suppression levels were suboptimal, and a phased release strategy was implemented. In Phase II, we released *Wolbachia* males into each of the four treatment clusters five times per week.

### Container-based male releases by hand

Throughout the project period, we identified areas within treatment clusters with consistently high female counts in AGOtraps. For some of these areas, vans were not able to drive through given the housing configuration, with only pedestrian access available such as in cluster ST02. In ST02, hand releases were conducted starting in June 2021 in an apartment complex where vans could only access facility edges through parking lots. Hand releases were added to courtyards that were enclosed on four sides by buildings. In PL01, hand releases were added to increase male density inside an apartment complex similar to ST02. In PL02, hand releases were conducted inside a cemetery. Hand releases began in PL01 and PL02 during the second week of August. The last hand releases were completed during the third week of November 2021 due to logistical constraints, approximately one month before project completion.

### Integrated vector management

The goal of this project was to measure the efficacy of *Wolbachia-*male releases as a tool for reducing mosquito populations in Ponce, PR, and ultimately disease transmission, so we chose to minimize integrated control measures. While the community education aspect of the project may have resulted in increased mosquito awareness among residents, it was unlikely to have resulted in significant impacts on the wild population without more dedicated efforts. Other than a single application of larvicide to three subterranean water meter pits in ST02 and unknown levels of private pest control activity, no additional, systematic chemical applications were made in the treatment or control clusters during the project period.

### Longevity assay

Life span of male mosquitoes was measured using a cage-based longevity assay as described previously [21]. Briefly, samples of ~100–200 packed and shipped male mosquitoes with *Wolbachia* and held-back males were released into a cage either in Ponce on the morning when they would have been released in Ponce (“packed and shipped”), or at Verily’s facility (“held-back” or unshipped controls). Males were provided with water in the cage, but no sugar, and dead males were counted every morning until all had died. Average lifespan was calculated as the average number of days it took for males to die after being released into the cage.

### Mating competition assays

Male competitiveness was measured using cage competition mating assays. Wild-type males and females from a colony established from locally collected eggs were reared in insectary conditions using standard protocols. *Wolbachia-*male mosquitoes were either sampled from production batches and held at Verily’s facility in California or sampled from shipments to Ponce. For each cage assay,10 3–5 day old wild-type females, 10 3–5 day old wild-type males, and 10 *Wolbachia-*males were placed in a medium BugDorm (30cm^3^, BioQuip) cage and allowed to mate for one hour. We have found that one hour is enough time for 10 females to be mated while minimizing multiply-mated females, providing a reliable competitive measure. Females were then placed individually in fly vials with seed germination paper and given a blood meal to elicit oviposition. All females were included in the analyses except those that did not lay eggs, were found to be uninseminated, or perished. Eggs were given 72 hrs to mature in insectary conditions and then flooded for hatching [38]. Any egg batch that produced at least one larva was considered wild-type mated and male competitiveness was calculated as the proportion of females that were mated to *Wolbachia-*males in each cage.

### Mark-release recapture assessment

We conducted two mark-release recapture assessments to evaluate dispersal distance and survival of packed and shipped *Wolbachia* males. We placed 34 BG Sentinel traps baited with BG lures in an evenly spaced grid extending out to 350m in a residential neighborhood within the treatment cluster SA01. BG traps were turned on ~24 hours after each release and serviced every 24 hours for five days. Fan speed and any trap issues were noted for each collection. All *Ae*. *aegypti* males were returned to Verily and tested for *Wolbachia* DNA using a LAMP assay as described previously [21]. Corrected recapture rates and daily survival were estimated by fitting a non-linear model, and confidence intervals were calculated using simulations, both based on the method of Buonocorsi et al [41]. Mean distance traveled was calculated by first correcting for variable trap density [42,43] at increasing distances from the release point and taking the mean of recapture estimates in 25m annuli.

### Measuring impact on wild populations

Since female mosquitoes take blood meals and are responsible for transmitting disease, wild female densities are the primary endpoint used to assess the impact of the treatment. We expected that if the intervention had no effect, female densities would be equivalent between treatment and control clusters. If the intervention had an effect, female densities would be significantly lower in treatment clusters compared to control clusters. For Phase I, we estimated the preliminary impact of the male releases on the wild population of female *Ae*. *aegypti* by comparing all treatment clusters in aggregate to all control clusters in aggregate using Abbott’s formula [31], and the 95% confidence interval was calculated using 10,000 bootstrap samples.

For Phase II, we incorporated the male releases in a model including additional parameters to better estimate the impact of the releases. Trap counts of female *Ae*. *aegypti* mosquitoes (per trap-week) were regressed on collection week using a generalized additive model, assuming a Poisson distribution for weekly trap counts and using a log link function[44]. The effect of week was represented by a polynomial spline of order three; treatment, temperature, relative humidity, and days since the last full trap service were included as terms in the linear operator. On the link scale, smooth functions of time (averaged across nested traps and sites) were estimated for (log)counts in the treated group and for (log)counts in the control group. These smooth functions were evaluated at the means of the other continuous variables in the model. To obtain the ratio of counts in the treatment group to the counts in the control group, the differences between the treatment curve and the control curve were computed at each time. Exponentiated, these differences become ratios on the response scale.

Using the estimated covariance matrix of the parameter estimates (including basis function parameters), standard errors for differences at each time point were computed. Residual degrees of freedom were used to find the t-distribution critical value, and confidence intervals for the differences were computed as point estimate ± t*se. The results were exponentiated to obtain confidence intervals for the ratios at each time point.

All data analysis was conducted in R v4.2.1[35], including R packages mgcv[45], RgoogleMaps[46], gplots [47], and RColorBrewer [48].

### Overcompensation analysis

To test for ecological compensation and overcompsensation, we re-calculated several statistics from our trapping data. First, we re-calculated summary statistics from both adult and egg collections in rolling one-month windows. To calculate the change in suppression, we calculated suppression by comparing one month windows of female trap counts as described above, then calculating the difference between consecutive windows. Other statistics were calculated as described above. Second, we tested for significant correlations between statistics using the cor.test function (method = ‘pearson’) in R.

## Results

### Incompatible mosquito shipping and releases

**Fig 2:**
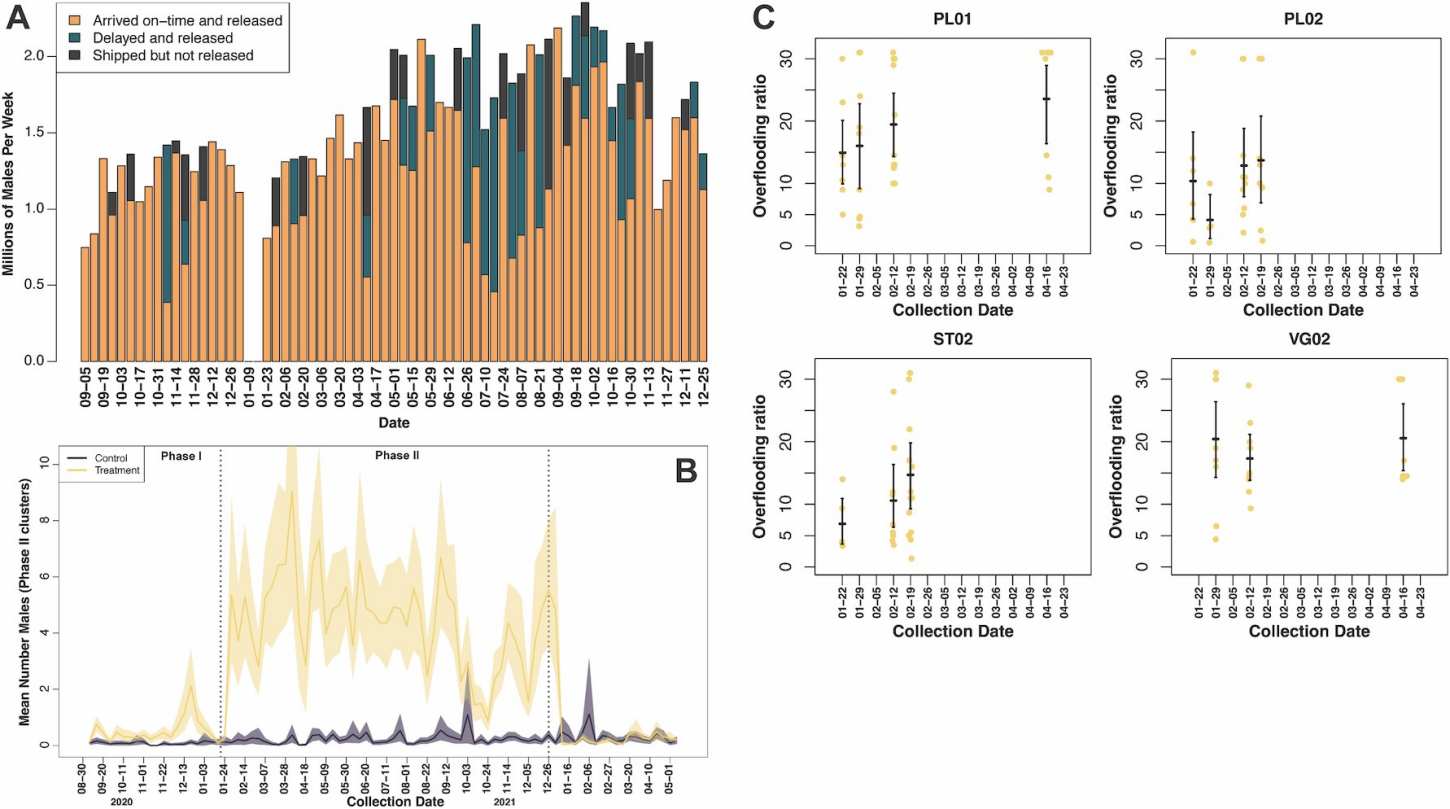
Male mosquitoes with *Wolbachia* released in COPA clusters, September 2020–December 2021, Ponce, Puerto Rico. A) Weekly male shipment totals (in Millions) shown on y-axis, with shipment date on the x-axis. Each bar is one week, summarizing five shipments per week. The orange bars show the total number of males that arrived on-time and were successfully released. The green bars show the number of males that arrived between 24–48 hrs that were released. Black bars show the number of males from shipments delayed beyond ~48 hrs were not released. B) The mean number of *Ae*. *aegypti* males collected in AGO traps in 2021 calculated across 4 treatment clusters (yellow) and all control clusters (grey). Shaded area shows 95% bootstrap confidence intervals. The vertical dotted lines show when releases began and ended in 2021. C) Overflooding ratio shown on y-axis from BG Sentinel collections according to dates on x-axis, with the cluster name shown for each sub-panel. Each dot is one BG trap collection. The middle horizontal line shows the mean for a weekly collection, with the 95% confidence interval shown with the vertical bar.

*Wolbachia*-male *Ae*. *aegypti* were mass-reared in South San Francisco, California, chilled, packaged, and shipped to Puerto Rico via commercial airline cargo shipping services using novel packing and shipping technology. Across both years of the project, a total of 107.6 million males were shipped, with weekly totals starting at 0.75M during the ramp-up in 2020 and peaking at 2.35M males per week in 2021 (Fig 2). Various constraints in the shipping and regulatory inspection pipeline limited shipments to five out of seven days per week. Successful SIT or IIT interventions require a consistent presence of released males, so disruptions to release schedules can cause reductions in efficacy, especially when the disruptions are clustered in time. The males were delivered in 345 shipments with an overall on-time success rate of 79%. We experienced many delays and complications related to the ongoing COVID-19 pandemic during the project, resulting in 53% of weeks during the project period experiencing some delay, with 9 weeks seeing on-time rates less than 50%. In some cases, shipments were delayed or canceled resulting in delayed or canceled scheduled releases, and ultimately resulting in the loss of 7.6M males during the project that were never released.

### Phase I: Releases in 19 clusters

During the first phase of the releases from September to December 2020, we shipped 22.31M males, and we successfully released 20.76M males across all 19 treatment clusters. We released an average of 56 (range 36–70) males per household per week (or 1,024 males/ha/week range 665–1281). Using the marginal male capture efficiency of AGOs, we detected a modest but significant increase in male *Ae*. *aegypti* in the treatment areas relative to controls (S3 Fig). However, after 10 weeks of releases, we observed only a minor impact on the wild female population with a preliminary reduction of 15% (95% confidence interval, 4–24%) when comparing all treatment clusters in aggregate to control clusters. We concluded that our *Wolbachia-*male release rate was insufficient to achieve strong reductions in the wild population.

### Phase II: Step-wise releases

**Fig 3:**
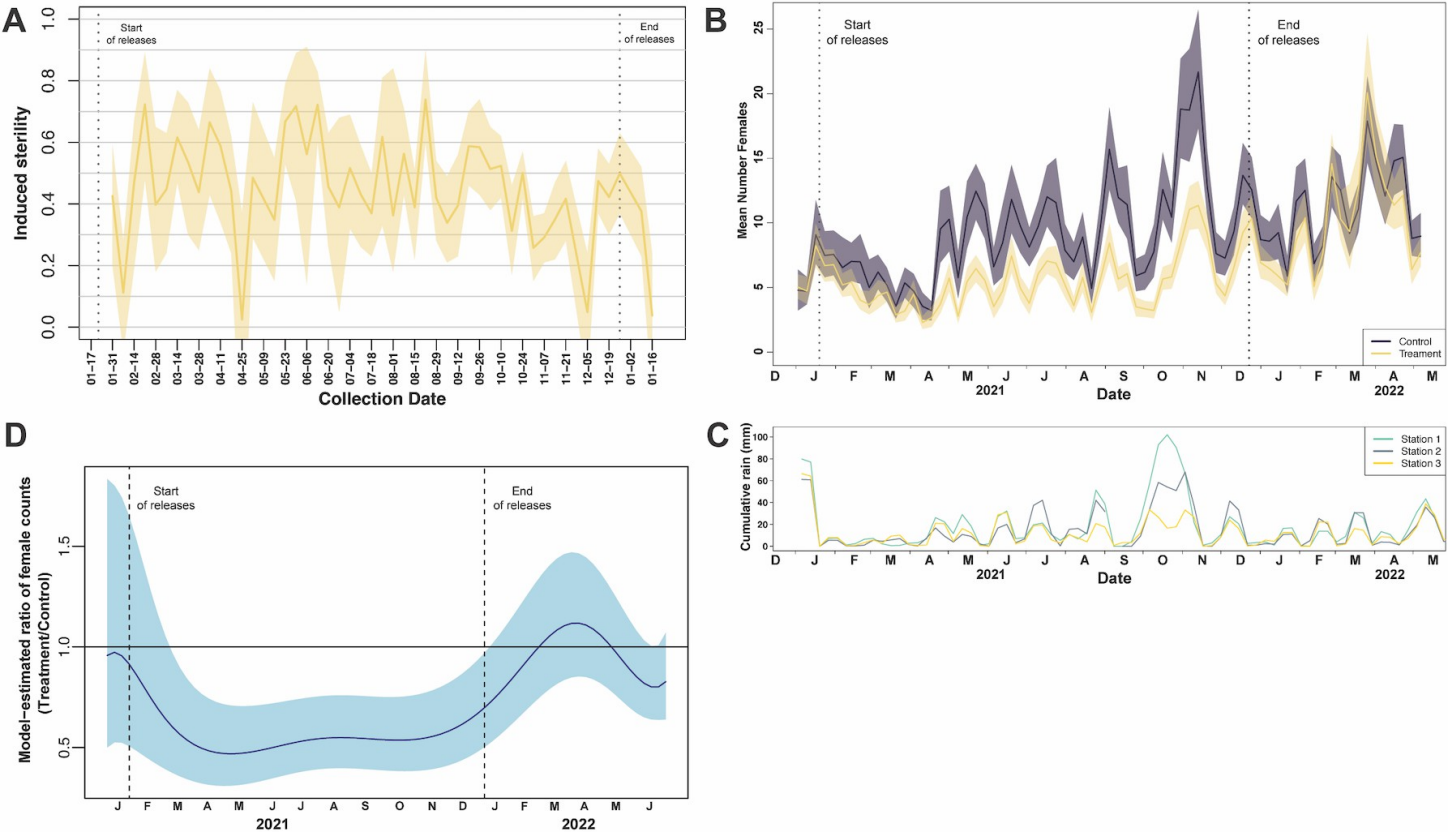
A) Induced sterility calculated as the scaled difference between treatment clusters aggregated hatch rate vs. all control clusters aggregated scaled by the control clusters hatch rate. Shaded area shows the 95% bootstrap confidence interval. Dotted lines show when releases started on left and ended on right. B) Mean number of female *Aedes aegypti* per trap per week in treatment clusters versus control clusters. December 2020–May 2022. Ponce, Puerto Rico. Dotted lines are the same as panel A. C) Cumulative rain over 21-day windows plotted on the same weekly schedule as in B for three weather stations in Ponce. D) Ratio of female *Aedes aegypti* mosquitoes in treatment clusters (n = 4) to control clusters (n = 4), Ponce, Puerto Rico, January–December 2021. Shaded area shows point-wise 95% confidence region. Dotted lines same as panel A.

After a brief three week pause in releases after Phase I, we began Phase II releases in the four treatment clusters in the last week of January 2021. From January until December 2021, we released a total of 79.71M *Wolbachia-*males (Fig 3). We started releases by applying the same average release rate to each of the 4 clusters at a rate of 375 males/household/week (or 6,338 males/ha/week).

During Phase II releases, we monitored male densities in the treatment clusters in several ways. First, we found the mean number of male *Ae*. *aegypti* in the AGOs rose to ~5 per 7-day collection period compared to ~0.1 in the control clusters. Second, we added BG sentinel traps that are designed to also capture adult male mosquitoes and found that the mean number of males increased to an average of 39 males per collection in the treatment clusters compared to an average of three in the control clusters (Fig 2C). We also spot-checked the overflooding ratio more directly by testing males from treatment clusters for *Wolbachia* DNA using a LAMP assay and found that the ratio (*Wolbachia*:wild males) varied substantially among trap collections, with ratios close to 1:1 in some collections while we detected only *Wolbachia*-males in others (Fig 2D). The mean overflooding ratio was generally high, with means greater than ~15:1 (*Wolbachia*: wild males) in all three sampled weeks in PL01 and VG02, while the means were more variable in PL02 and ST02. Taken together, these results suggest that consolidating the releases into four clusters resulted in substantial increases in male densities and overflooding ratios closer to our target levels.

If the released *Wolbachia*-males were successfully penetrating the landscape and mating with wild females, we would expect eggs collected from these landscapes to hatch at a lower rate than those collected from control clusters. To test for this effect, we added ovitraps to each treatment and control cluster. We compared the proportion of eggs that hatched in treatment clusters to that of the control clusters and found that our male releases induced significant levels of sterility throughout Phase II release period (Fig 3A). Except for several outlier weeks, induced sterility levels remained approximately 0.5 from the start of releases until the end of August, at which point induced sterility levels began trending downwards (Fig 3A), possibly consistent with either a drop in mating pressure from the *Wolbachia-*males, or an increase in migration from untreated areas nearby.

The goal of the *Wolbachia*-male releases was to reduce the number of wild female mosquitoes that could contribute to disease transmission. We monitored female densities in the treatment and control clusters using AGOs throughout the project period and using BG sentinels from February through August 2021. Prior to the start of releases, the mean number of females in the treatment and control clusters were well correlated and both fluctuated substantially, sometimes by 2-fold over the span of two weeks (S4 Fig), emphasizing the importance of considering population density changes in the control clusters and other environmental factors when evaluating impact of the *Wolbachia* releases. After releases began, the mean number of females in both the treatment and control clusters decreased on average for the first 12 weeks of releases (Fig 3B). Although the populations in the treatment clusters decreased at a faster rate than those in the control clusters, the impact of the *Wolbachia*-male releases is confounded by a drop in mosquito populations in the region, likely due to a marked lack of rain during these months (Fig 3C). Following the first sufficiently consistent rains of the year in mid-April, populations in the control clusters and to a lesser extent the treatment clusters rebounded, suggesting the presence of a substantial egg bank in these communities. From May until October, we observed a repeating saw-tooth pattern in both treatment and control clusters, followed by a large increase in all populations starting in mid-October, consistent with local rain patterns driving large hatching events (Fig 3B).

To best estimate the effect of the *Wolbachia-*male intervention while accounting for environmental and operational effects, we used a Generalized Additive Modeling approach and found that the weekly counts of female *Ae*. *aegypti* mosquitoes in the four treatment clusters were 51% (95 CI 37–71%) of the counts in the control clusters (Fig 3C). Maximum reduction was achieved at the beginning of May 2021 when the rains began (mean of 4.1 and 8.5 females in treatment and control, respectively), and the reductions remained around 50% for most of the Phase II release period. Once releases stopped, the average weekly female mosquito counts in the treatment clusters increased steadily, reaching the same levels as the control clusters in about 2 months. These results suggest incompatible male releases had a clear suppressive effect on the wild population. However, the reduction observed in the four Phase II clusters was lower than the target level required to bring the *Ae*. *aegypti* population to a level where viral transmission is reduced [49,50]. Below, we describe various adjustments made to the release strategy as well as attempts to identify opportunities to improve the strength of the intervention.

### Adaptive release strategies

**Fig 4:**
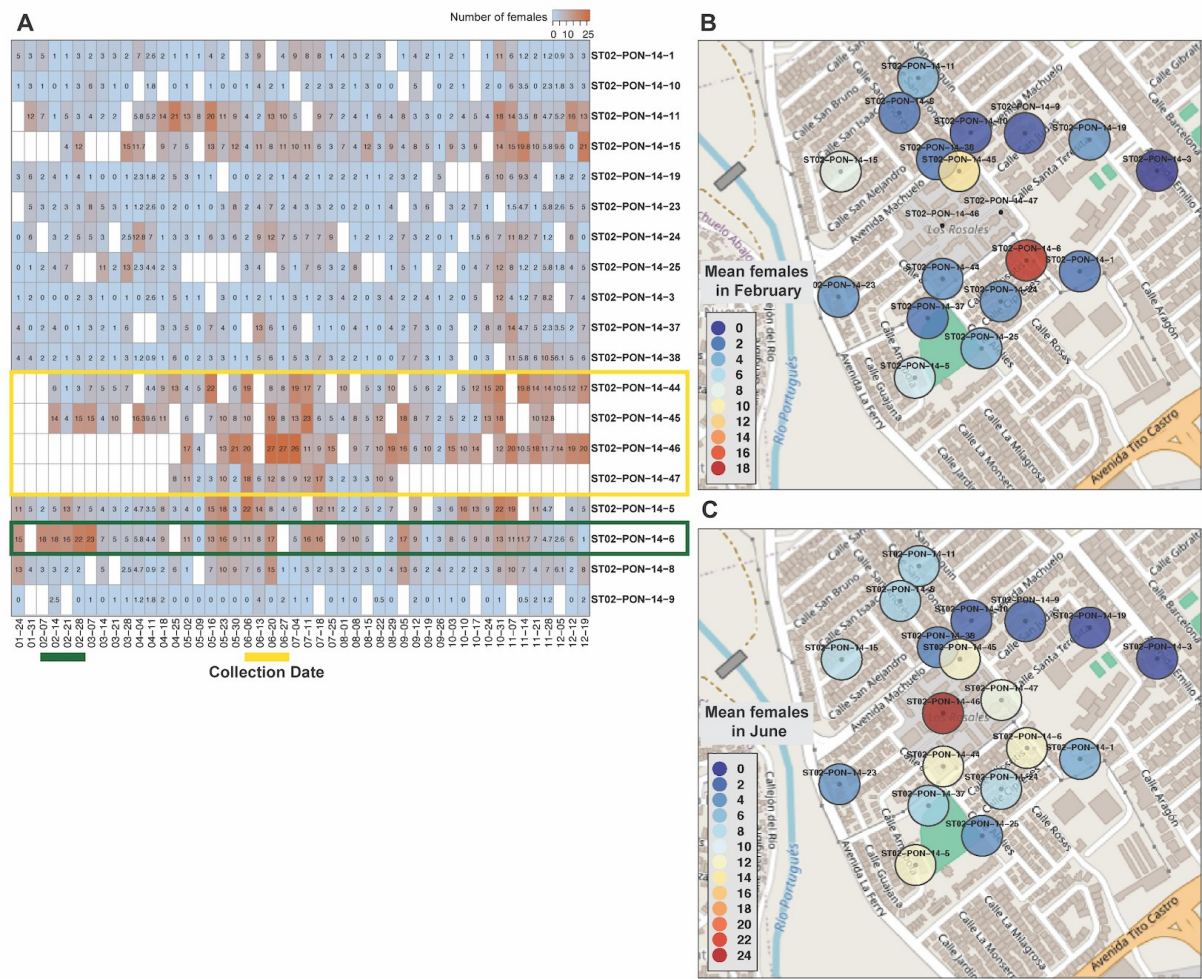
Hotspot areas in cluster ST02. A) Heatmap showing *Ae*. *aegypti* female counts by trap in rows and collection week in columns with colors according to key above. Trap labels to the right of heatmap. Actual counts are labeled in each cell. Specific traps discussed in text shown with yellow and green boxes. Some traps were added after the releases began. Yellow and green bars below indicate weeks used for means shown in panels B (green) and C (yellow). B) Four-week mean number of females in April 2021 (green bar under heatmap) in traps in ST02 according to legend colors showing outlier collection numbers in trap 14–6. Weekly collections for this trap shown in panel A. C) Same as B but for June 2021 (yellow bar under heatmap) showing high female means in the center of the cluster corresponding to an apartment complex. Map source: OpenStreetMap.org (CC BY-SA 2.0). Open Street maps were labeled in R using the Rgooglemaps package. https://www.openstreetmap.org/#map=14/18.01318/-66.60985.

As releases were ongoing, we noted that female densities were spatially variable within the treatment clusters, so we made a series of adjustments to focus releases in these areas. In one case, we observed a single AGO trap (ST02-PON-14-6) in ST02 that consistently captured higher female counts than other traps in this cluster (Fig 4). We increased the release rate from the van on February 25, 2020, at all release points within ~50m of the trap location. The female densities in this trap were reduced by more than half three weeks after this adjustment. In the same cluster, ST02, we noted that traps inside or on the edge of a residential apartment complex consistently captured high numbers of females. We had been using the release van to treat this area by treating the adjacent roads and also releasing in parking lots that enter the edge of the property. To improve our monitoring of the wild population in this complex we added two AGO traps inside the complex and confirmed that the wild population was high. After confirming the need for additional treatment, we began releasing inside the complex by hand releasing from small containers in the last week of June at designated locations inside of the courtyards that were blocked from the exterior streets where vans could release. Similar to the impact we observed at ST02-PON-14-6, the number of females in traps in and around the residential complex began to decrease ~3 weeks after the addition of hand releases (Fig 4A, green box). We similarly added hand releases to a large cemetery and a residential complex, both in PL01, and made a number of smaller adjustments to van release rates throughout all four treatment clusters, in an attempt to improve control of problematic breeding locations detected by traps, and thus suppression overall.

### Mosquito quality assessments

**Fig 5:**
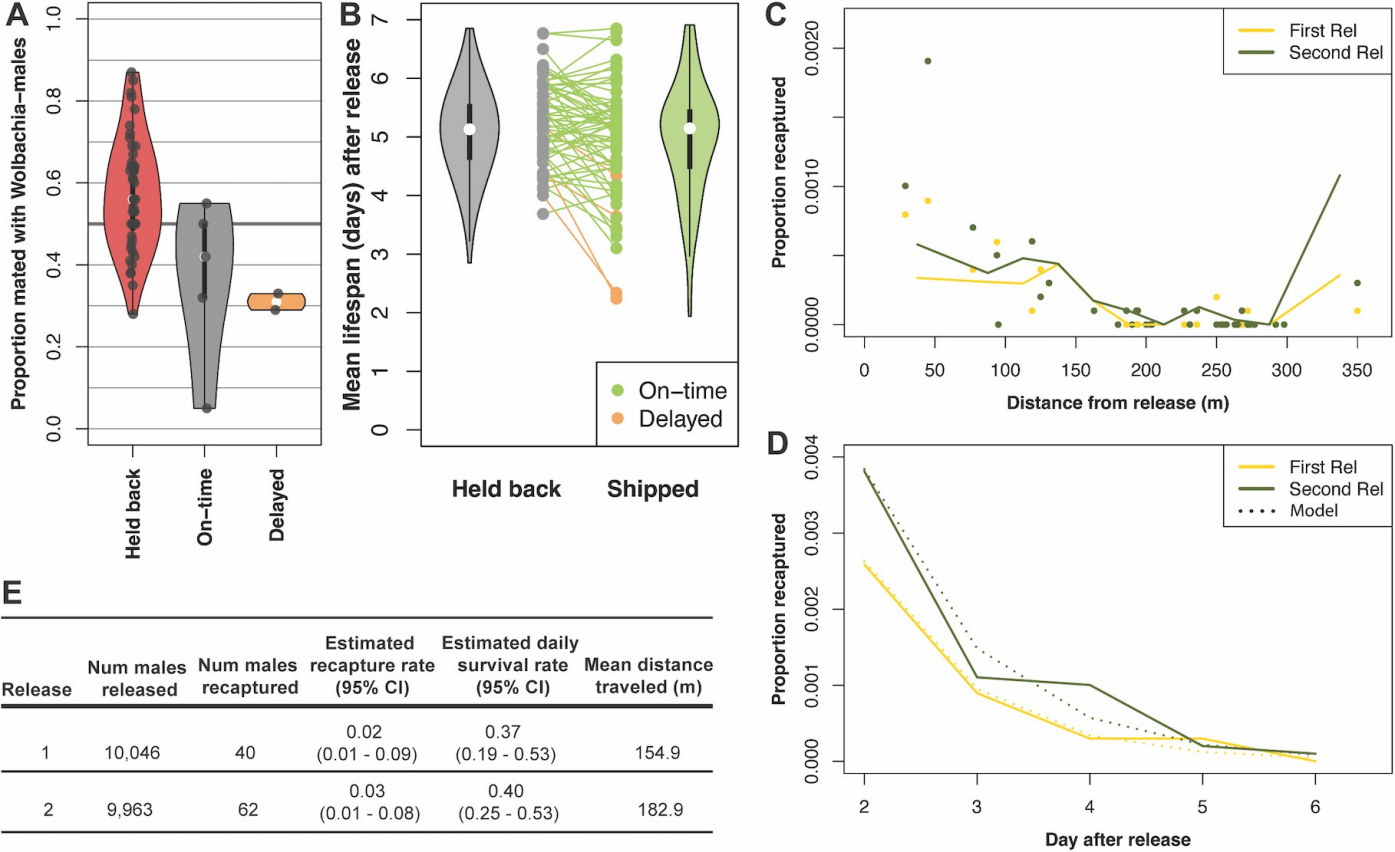
Male quality assessments. A) Mating competitiveness cage assays. Each dot indicates the proportion of females mated with a *Wolbachia*-male in competition with wild-type males. Violin plots show median of distribution with white dot and quartiles. B) Reaction norm plot comparing mean lifespan for samples of males from the same batch assayed in San Francisco (held back) or in Ponce (shipped), either on-time or delayed according to legend. Violin plots summarize distribution of held back and shipped samples separately. C) Proportion recaptured (y-axis) in mark-release recapture (MRR) study as a function of distance from release point (x-axis) for two replicates according to legend. Each dot shows the total collected for a trap, and the lines show corrected collection rate (see methods). D) Proportion recaptured (y-axis) in MRR study over days since release (x-axis). Solid lines show empirical data and dotted lines show best-fit model for each replicate. E) MRR summary statistics. Estimated recapture rate and estimated proportion daily survival rate refer to values inferred from model fitting with 95% confidence intervals calculated from simulations (see Methods). Mean distance traveled in meters after correcting for variable trap density (see Methods).

Reductions in male fitness related to shipping or other factors could limit their efficacy in the field, so we conducted a few assays to assess male quality. To test for large reductions in fitness related to the packing and shipping process, we conducted caged mating competition assays comparing *Wolbachia-*males, either held back or shipped, to wild-type males. If the two types of males were equally fit, we would expect approximately equal numbers of females mated to each type resulting in a mating rate of 0.5. We conducted a number of mating assays during the development stages of the shipping technology as well as over the duration of the project. We found that, although these assays are highly variable, *Wolbachia*-males that were held back before shipping competed for mates at an approximately equal rate to wild-type males (Fig 5), but mating rates of shipped males were reduced, with marked differences by shipping vessel type and if shipments were on time or delayed. At the beginning of the project in 2020, we made several adjustments to the shipping vessel and conducted mating assays for each prototype iteration. Mean male mating rates improved from 0.17 to 0.34 over the first three prototypes (v1-v3, S5 Fig) in our preliminary assays with simulated shipping conditions, but further improvements produced a fourth prototype (v3.1) that resulted in a mean mating rate of 0.44 (n = 4, S5 Fig). In the late stages of the project, we tested two additional modified versions of the shipping vessel and saw mixed results with one version (v3.2) resulting in mean mating rate of 0.29 (n = 4), while the other version (v4.0) resulted in a mean mating rate of 0.58 (n = 9). We used v3.1 to ship *Wolbachia*-males for most production shipments during the project and found a mean mating rate of 0.37 (n = 5) after shipping (Fig 5). We also assayed two samples from a single delayed shipment batch and found reduced mating rates with the *Wolbachia*-males (mean = 0.31). Cage-based mating assays provide coarse measurement of fitness, but we detected a clear impact of the packing and shipping on male fitness. It remains unclear how this fitness cost translated to field efficacy.

Throughout the project, we measured male longevity of samples held back at the Verily production facility as well as samples after both on-time shipping and delayed shipping to Ponce. We compared samples taken from the same rearing batch and found that on-time shipped longevity was significantly lower than longevity for held back samples that were not shipped (Wilcoxon signed rank test *P =* 0.0379, n = 75 batches, Fig 5), although the difference between means was small (5.06 days for shipped vs. 5.31 days for held back), suggesting only minor impacts overall of packing and shipping on male longevity under laboratory conditions. We only assayed a small number of samples from delayed shipments, but the impact appeared stronger for these samples with a mean longevity of 3.14 (n = 4 batches) compared to 4.54 for held back samples from the same batch, which is consistent with our observations that males from delayed shipments appeared sluggish and unfit. Fig 5B reveals longevity estimates for shipped males that both increase and decrease underscoring the noise associated with this assay, and the overall reduction of the shipped class is small but significant under these conditions. It remains unclear how the differences we measure in the lab translate to the field, but it is clear that packing and shipping had at least some negative impact on male fitness.

Mark-Release-Recapture (MRR) assessments can provide more realistic estimates of longevity and flight performance in the field, so we conducted a series of two MRRs in a community not involved in active suppression releases. We recaptured a very small proportion of the released males for both replicates (rep 1 = 0.004, rep 2 = 0.006). We placed BG Sentinel 2 traps out to 350m from the release point and estimated mean distance traveled of 155m and 183m for replicate 1 and 2, respectively, after correcting for variable trapping effort (Fig 5C and 5E). To jointly estimate a corrected recapture rate and daily survival, we fit a non-linear model (Fig 5D) to the recapture data [41] and obtained very low daily survival rates (0.37 and 0.40, Fig 5E) and corrected recapture rates around 2% and 3% for replicates 1 and 2, respectively. Daily survival rates of ~0.4 suggests that only 40% of males survive each day resulting in very low male densities after only two days post release. Although the recapture rates were very low, data from two MRR replicates are consistent with significant negative impacts of the shipping on the daily survival of *Wolbachia-*males, likely limiting efficacy of the intervention.

### Ecological compensation of the wild population

**Fig 6:**
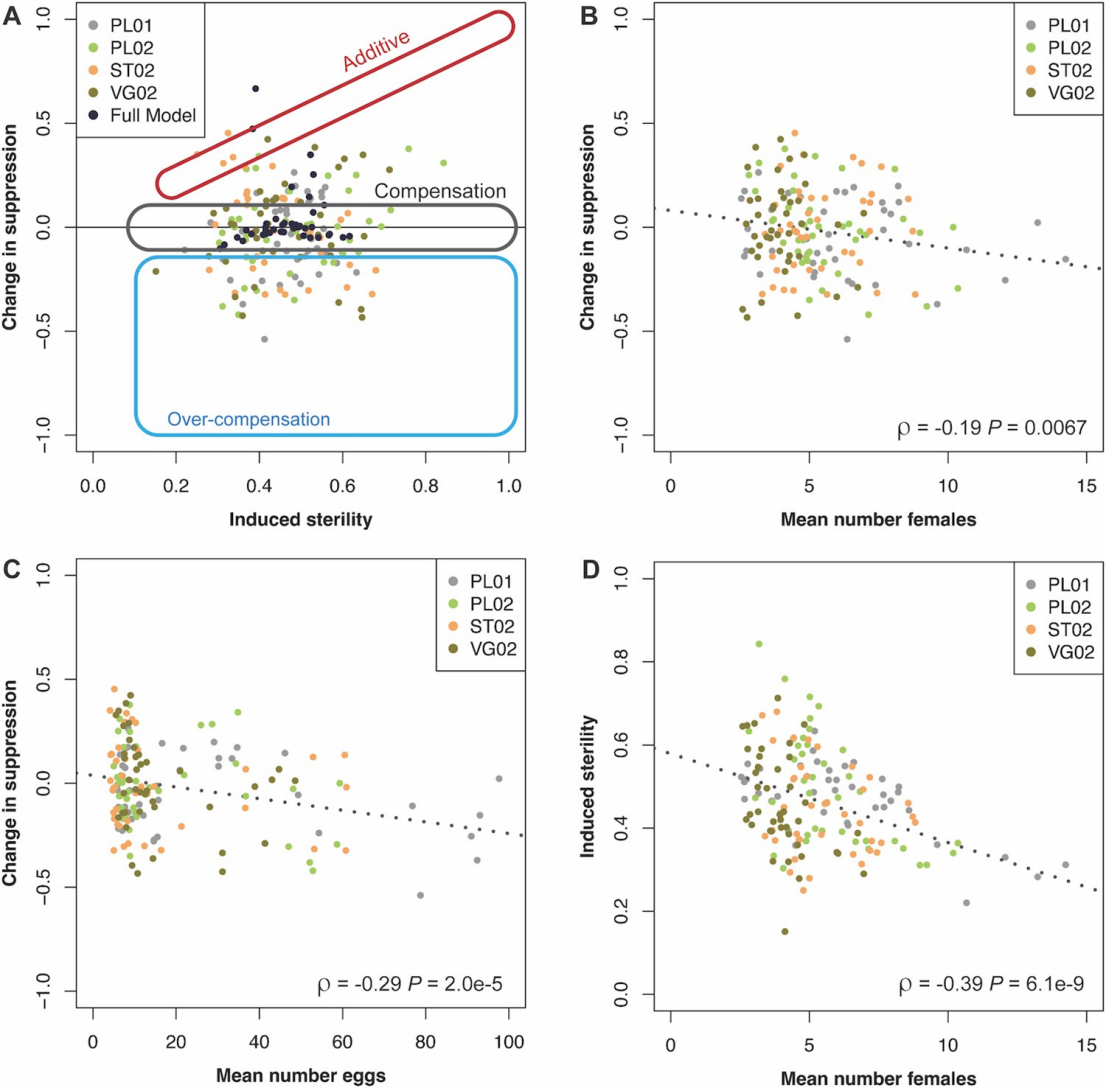
Evidence for ecological compensation and overcompensation. Each dot represents the mean for a four-week window for each Phase II treatment cluster according to the legend. A) Change in suppression calculated using before-after-control-impact comparing windows separated by three weeks. Analysis of fitted Generalized Mixed Model shown in black. Ovals show regions where values under Additive, Compensation, and Over-compensation models would be expected. B) Impact on suppression as a function of mean female densities at the time of application. C) Impact on suppression as a function of mean egg densities at the time of application. D) Induced sterility as a function of female densities in the same window. Dotted lines show best-fit line and results of Pearson’s correlation test shown in bottom right.

Another possible explanation for limited suppression is that our intervention produced a compensatory or overcompensatory response, where survival to adulthood is either unchanged or increased, respectively, after extrinsic mortality, such as predation or IIT interventions, is applied to the wild *Ae*. *aegypti* population[51,52]. Recent studies suggest that removal of *Ae*. container breeding mosquitoes at the embryonic stage, as is the case during IIT and SIT interventions, can relieve resource competition in the larval habitat resulting in higher survival to adulthood than without the intervention [53,54]. To test for evidence of compensation or overcompensation, we summarized induced sterility, mean female numbers, and suppression on a monthly basis and made several comparisons to ask whether our data fit expectations under these models. First, we compared the change in suppression (after three-week lag, calculated using Abbot’s formula, see Methods) to induced sterility using a Before-After Control-Impact (BACI) analysis at the cluster level and saw a large proportion of months consistent with compensation in all clusters where induced sterility was at least moderate with little to no impact on suppression three weeks later (Fig 6). A small number of months were in line with an additive effect, but a much larger proportion of months showed negative change in suppression despite moderate levels of induced sterility (Fig 6) consistent with over-compensation. Importantly, we used a GMM to infer suppression while accounting for other variables such as rain that are clearly important drivers of female densities, and comparison of BACI analysis using GMM model output with induced sterility levels provides support for a compensatory response throughout the project (Fig 6).

One of the recommendations for avoiding compensatory and over-compensatory effects during vector control interventions is to remove larvae, or induce sterility, when the population density is lower than normal [51]. To test whether this dynamic may have impacted our project, we compared the change in suppression to both female densities (*ρ* = -0.19; P = 0.0067, Fig 6) and egg densities (*ρ* = -0.29; P = 2.0e-5, Fig 6) and saw a significant correlation in both cases, suggesting less suppression at higher female counts and egg counts. Interestingly, we also compared the level of induced sterility to the mean number of females at the time of application and saw significant and negative correlation (*ρ* = -0.39; P = 6.1e-9, Fig 6). Together these data are consistent with a scenario where the impact of male releases was directly related to the wild population density as would be expected if release rates or male fitness were insufficient to compete with high density wild populations.

## Discussion

Limited vector control methods are available for use in Puerto Rico given the documented high insecticide resistance of local mosquitoes[14] and logistical challenges on the extended implementation of other effective methods like source control and mass trapping [49,50,55]. Robust evaluations of the efficacy of additional tools for *Ae*. *aegypti* control are needed, and in this project, we describe the implementation and results of the first release of *Wolbachia* incompatible *Ae*. *aegypti* mosquitoes in Puerto Rico and the Caribbean. Overall, an average suppression of about half (49%) of the mosquito population was achieved in the four treatment clusters included in Phase II of the project. Although the plan was to expand releases to all 19 treatment clusters from Phase I after each set was successfully suppressed, interim analysis of mosquito population reductions and the lack of dengue transmission led us to terminate release operations without further expansions. Large field assessments like this are complex with many factors contributing to the outcome, but several factors seem the most likely to be limiting suppression levels including high wild adult densities in the treatment areas, compromised male quality after packing and shipping, migration of mated females into the treatment areas, challenging population dynamics, and logistical and resource limitations to treating large, densely populated areas.

In practice, incompatible and sterile-insect releases should be implemented as part of an integrated vector control program with complementary interventions deployed before and during releases to improve both efficiency and efficacy [56]. In this project, the targeted communities did not undergo any routine, community-level mosquito control, and we chose to implement IIT without adding any supplemental control measures to best isolate the efficacy of the IIT in this setting. As a result, the density of the wild population was high at the beginning of releases with a substantial egg bank that responded vigorously to large rain events producing large numbers of adults that would be challenging for any control methods, but especially IIT since maintaining sufficient overflooding ratios would require significant increase in *Wolbachia* male mosquito release rates. We found that mosquito productivity was highly heterogeneous within the treatment clusters, with some traps consistently showing higher counts than those in neighboring areas. We made several adjustments to the release strategy including increasing van releases and adding hand releases to some areas, and saw some reductions in the number of females, but suppression at the cluster level remained limited. Overflooding ratios were high on average when we spot-checked, although highly variable among traps. The communities targeted in the treatment clusters included dense residential areas, apartment complexes, abandoned housing, commercial areas, and public spaces. Notably, the implementation of this intervention started in 2020, during the COVID-19 pandemic, when reduction in the number of available flights, flight delays, and staffing challenges were common. As a result, we experienced many delays and cancellations of male mosquito shipments leading to extended gaps in the release schedule that further limited released-male densities during certain weeks. It is likely that the male releases resulted in *Wolbachia*-male densities that were too variable in both space and time, leaving low density areas that were insufficient to achieve consistent pressure on the wild population, especially in the presence of any number of highly productive breeding sites that remained active throughout the project.

The ability to pack and ship incompatible or sterile male mosquitoes could drastically improve access to this technology by removing the requirement for mass rearing and sex-sorting infrastructure local to every new treatment area, but packing and shipping mosquitoes at scale adds significant risk to release programs. After several rounds of prototyping and improvements, the packing technology developed for this project allowed male transportation overnight with seemingly minimal impact on cage-based longevity, competitiveness, and flight ability, as long as the shipment was delivered within 24 hours of packing. Comparisons of longevity and mating competitiveness between shipped and held back males both pointed to a cost to the packing and shipping process, but the reduction was small in both cases. Results from the two MRR studies, however, point to a significant reduction of male fitness in the field that likely limited impact of the releases. Preliminary data and visual observations suggest that shipment delays extending male sedation past 24 hours had a significant impact on fitness. The dispersal kernel inferred from recaptures at increasing distances from the release point suggests that flight ability was in a range similar to other published MRR studies [32] with mean distance traveled >150m. However, we estimated daily survival to be 0.35–0.4, suggesting that only ~30–40% of released males survive each day after release leaving very low densities after only two days. We only recaptured ~100 males out of the ~20,000 released between the two replicates, which may reflect low sensitivity of BG Sentinel traps in this setting. But the shape of the curve impacts the inferred daily survival rate, so these results suggest that our males are not living as long as expected, despite their flight ability being unimpacted. The lab assays detected only small impacts of the shipping on fitness, but the MRR suggests that field longevity may be significantly reduced to a point where even three releases per week would struggle to maintain high male density in the field and operational disruptions leave large gaps in male densities. These results show that the shipping technology used for most of the shipments in the project had a significant impact on male fitness and therefore the viability of the intervention. In theory, male release programs could still be successful with lower fitness males, but higher release rates would be needed to compensate. In either case, additional improvements to the packing and shipping pipeline are needed and will likely improve shipped male quality. Newer versions of the shipping vessel such as version 4, already showed improved mating rates.

Migration of mated females and fertile males from nearby areas into treatment clusters has impacted other IIT and SIT studies[21] and may have had an impact on our results as well. To maximize statistical power for the epidemiological evaluation, the total available treatment area was subdivided into 38 clusters, necessarily creating many edge areas in the 18 treatment areas exposed to untreated areas nearby. Although the traps with consistently high female numbers were not always found at the edge of the treatment clusters, it remains possible that an influx of mated females and fertile males contributed to ongoing breeding in the treatment areas, ultimately limiting the levels of induced sterility, and therefore suppression, we could achieve. In practice, IIT and SIT should be implemented on larger areas to minimize the impacts of immigration.

IIT and SIT interventions remove mosquitoes at the embryo stage resulting in fewer larvae in the environment and potentially less competition for resources at the larval stage in containers, but the evidence is mixed as to how *Ae*. *aegypti* respond when extrinsic mortality is applied, and resource competition is relieved under various conditions [51–54]. It is notable that compensation and over-compensation effects, or greater production of adults when larvae are removed at some stage, are most likely when induced sterility levels are <0.5 and the target population density is high. Our results suggest that our intervention resulted in induced sterility levels around and often less than 0.5 throughout the project. We summarized our data by month and saw evidence most consistent with a compensatory effect. The strength of the effect can be modulated by other factors including resource availability and temperature [51] but we have no data on ecological conditions in larval habitats in Ponce, so it is difficult to develop clear expectations. Importantly, cumulative rain patterns are strong drivers of changes in mosquito populations in Puerto Rico [55]. We included an analysis of compensation using levels of suppression inferred from our GMM analysis that accounts for environmental factors and still see evidence consistent with compensation, but tests for compensation and over-compensation would be improved by incorporating environmental variables more directly. Assuming that compensation and over-compensation are possible during IIT and SIT interventions, two of the key recommendations for avoiding these effects are to apply very high (>70%) induced sterility through high and consistent overflooding ratios and apply the intervention when the population density is low, either seasonally or from complementary interventions [51]. Our data suggest that although releases started during the low mosquito season, additional interventions as part of integrated vector management are needed in tropical areas with robust mosquito populations. The possibility that the impact of our *Wolbachia*-male releases was limited by compensatory responses at the larval stage is intriguing, but more work is needed to robustly test this theory.

Adoption of new and effective technologies to control *Ae*. *aegypti* and other vectors of human disease is critical for slowing disease transmission. Broad approval and funding for such technologies is largely gated by the need for data showing significant epidemiological impact from two or more randomized controlled trials, as required by WHO [57]. Such studies are expensive, logistically complex, and dependent on virus transmission patterns, which can be highly variable over time. Our project underscores how unforeseen circumstances, in this case the absence of dengue transmission likely related to COVID-19 societal disruptions, can derail multiple years of planning, participant recruitment, and data collection. The impact of our *Wolbachia-*male intervention was modest and limited to only four of the 19 treatment clusters; additionally, we were not able to analyze the effect of mosquito population reductions on dengue transmission given the very low circulation of the virus in Ponce during the project period. This work highlights many of the challenges of conducting large field trials intended to measure entomologic and epidemiologic outcomes. However, the project successfully reduced the female *Ae*. *aegypti* population by half, identified important factors for the feasibility of shipping *Wolbachia*-male mosquitoes, and highlighted the importance of using an integrated approach to reduce mosquito populations. Future directions will include evaluation of improved mosquito shipping containers, the assessment of the effectiveness of the implementation of simultaneous vector control interventions and the investigation of more feasible ways to evaluate their epidemiologic outcomes, and continued investment in economically sustainable vector control tools to reduce *Aedes*-borne diseases.

## Supporting information

S1 Fig Mean number of female Ae. aegypti during pre-release phase from January to the end of August 2020 presented as a 3-week trailing average. AGO traps continued to be added and moved during this period with trap placements finalized by the end of pre-release period. Clusters chosen to receive treatment are presented in top panel and untreated control clusters presented in bottom panel.
(TIF)

S2 Fig Transportation logistics for the releases of male mosquitoes with *Wolbachia* in Ponce, Puerto Rico, 2020–2021. (TIF)

S3 Fig The mean number of *Ae*. *aegypti* males collected in AGO traps in 2020 calculated across all 19 treatment clusters (yellow) and all control clusters (grey). Shaded area shows 95% bootstrap confidence intervals. The vertical dotted line shows when releases began in 2020.
(TIF)

S4 Fig Mean number of female *Aedes aegypti* per trap per week in treatment clusters aggregated (n = 19) versus control clusters aggregated (n = 19) from January to December 2020. Shaded area indicates 95% bootstrap confidence interval. Dotted line shows when releases began in treatment clusters.
(TIF)

S5 Fig Mating assays for five prototype versions of the shipping vessel. For each assay, shipping was simulated by first packing Ae. aegypti wAlbB *Wolbachia* males under the normal protocol, but then placing the males inside the prototype shipping container. Shipping duration ranged between 20 and 24 hours.
(TIF)
