# Supplementary figures and images for: Incompatible Aedes aegypti male releases as an intervention to reduce mosquito population—A field trial in Puerto Rico
Source: PLoS Negl Trop Dis. 2025 Jan 21;19(1):e0012839. doi: 10.1371/journal.pntd.0012839 (PMC11785262; doi:10.1371/journal.pntd.0012839)

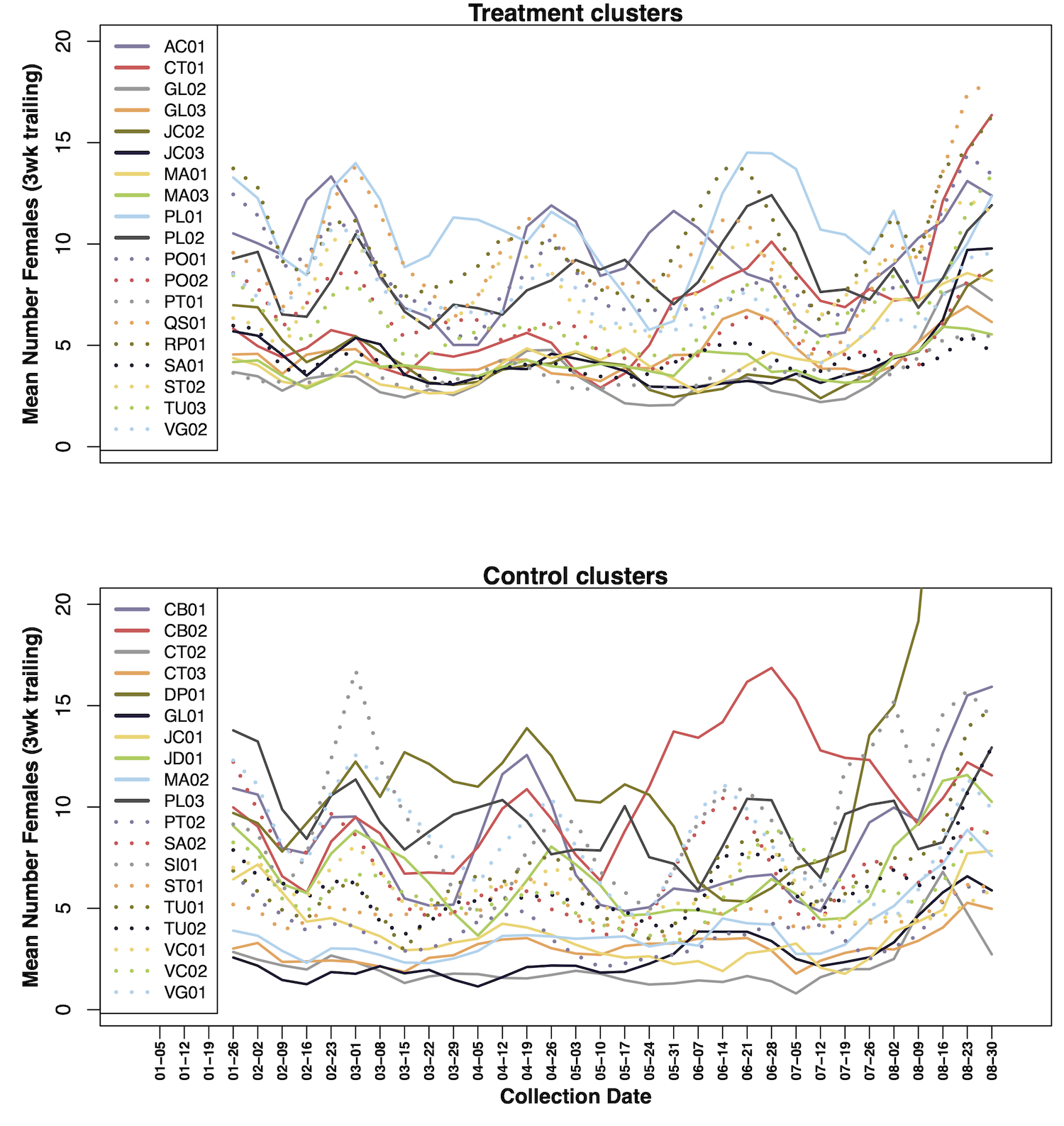

Supplement: S1 Fig — AGO traps continued to be added and moved during this period with trap placements finalized by the end of pre-release period. Clusters chosen to receive treatment are presented in top panel and untreated control clusters presented in bottom panel. (TIF) [file pntd.0012839.s001.tif]

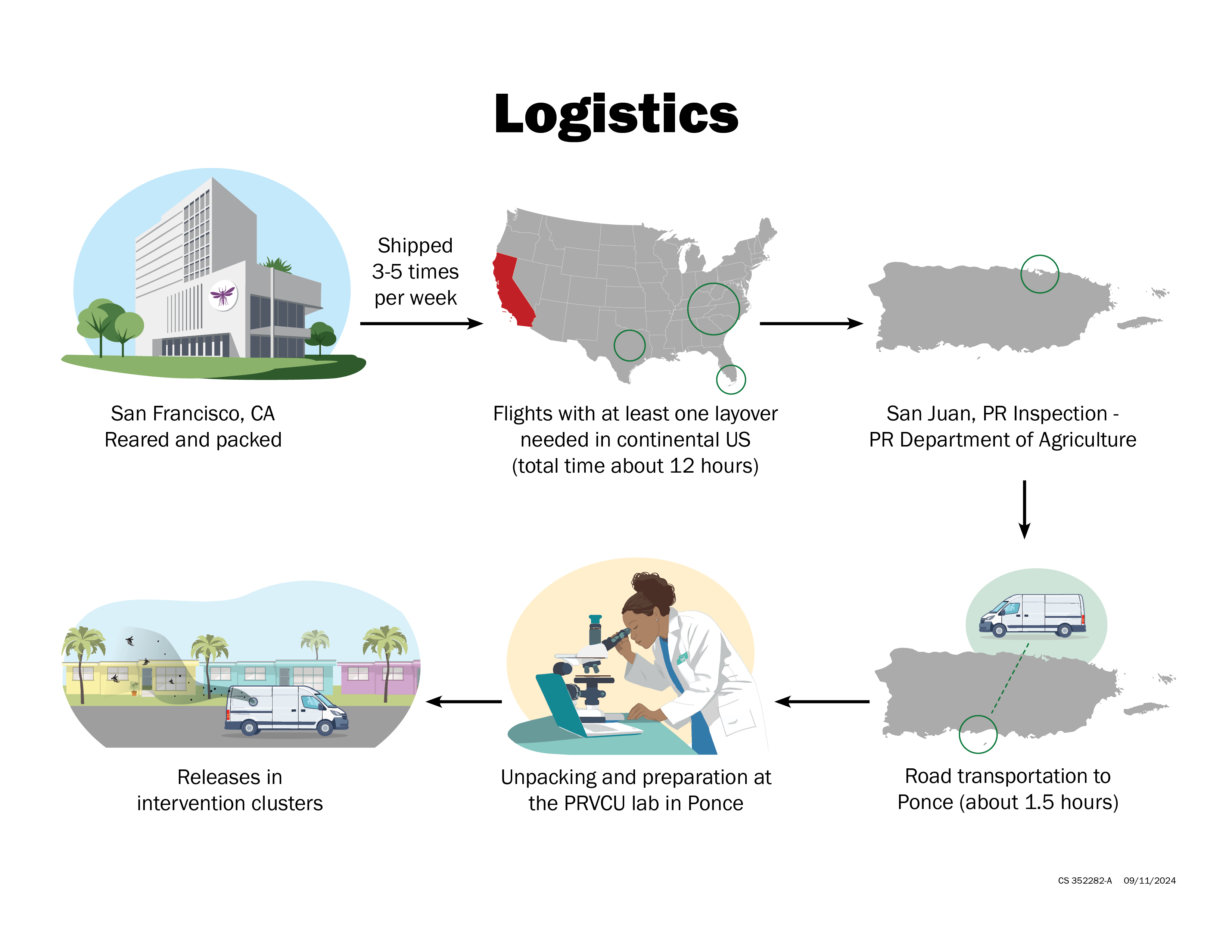

Supplement: S2 Fig — (TIF) [file pntd.0012839.s002.tif]

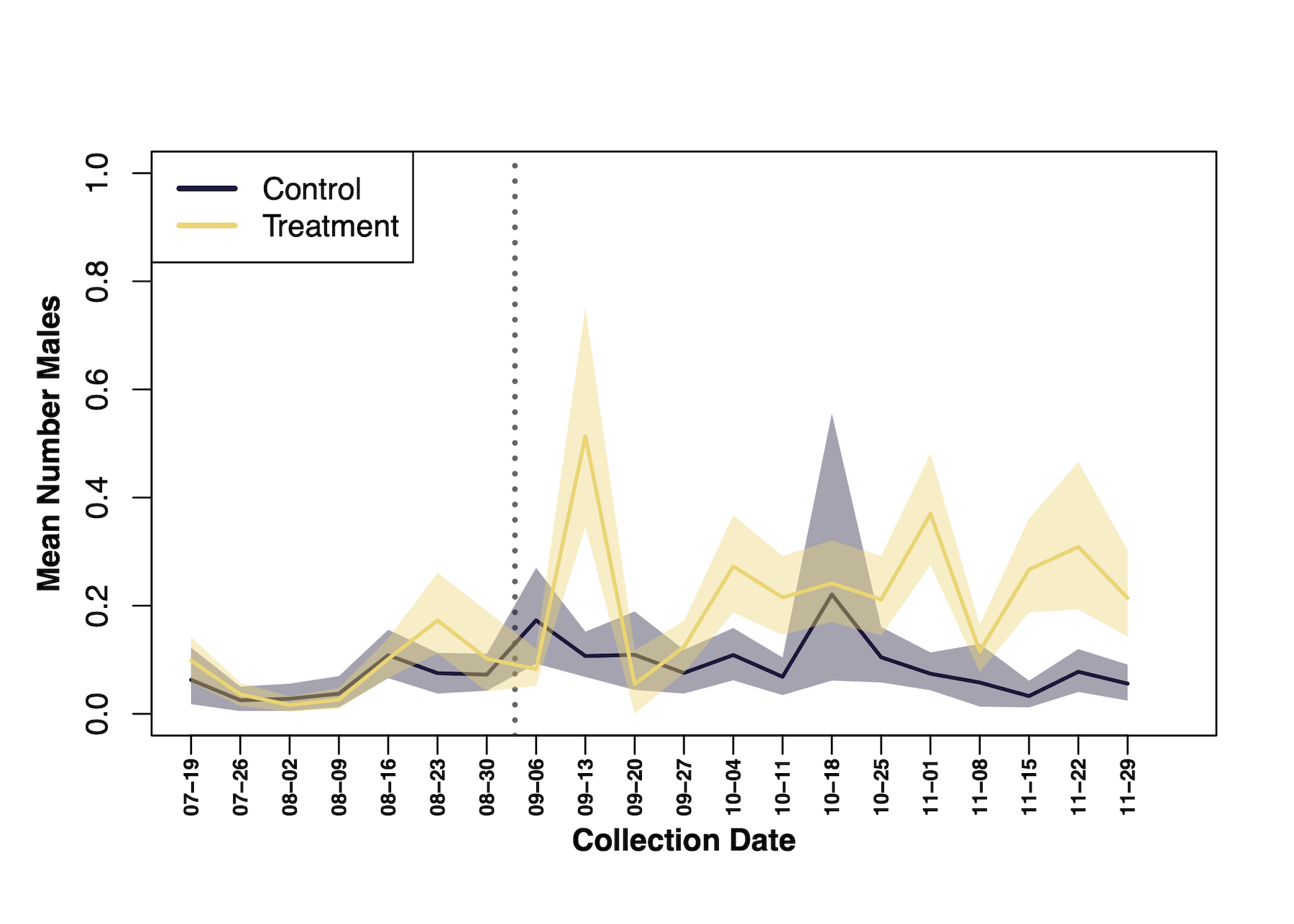

Supplement: S3 Fig — Shaded area shows 95% bootstrap confidence intervals. The vertical dotted line shows when releases began in 2020. (TIF) [file pntd.0012839.s003.tif]

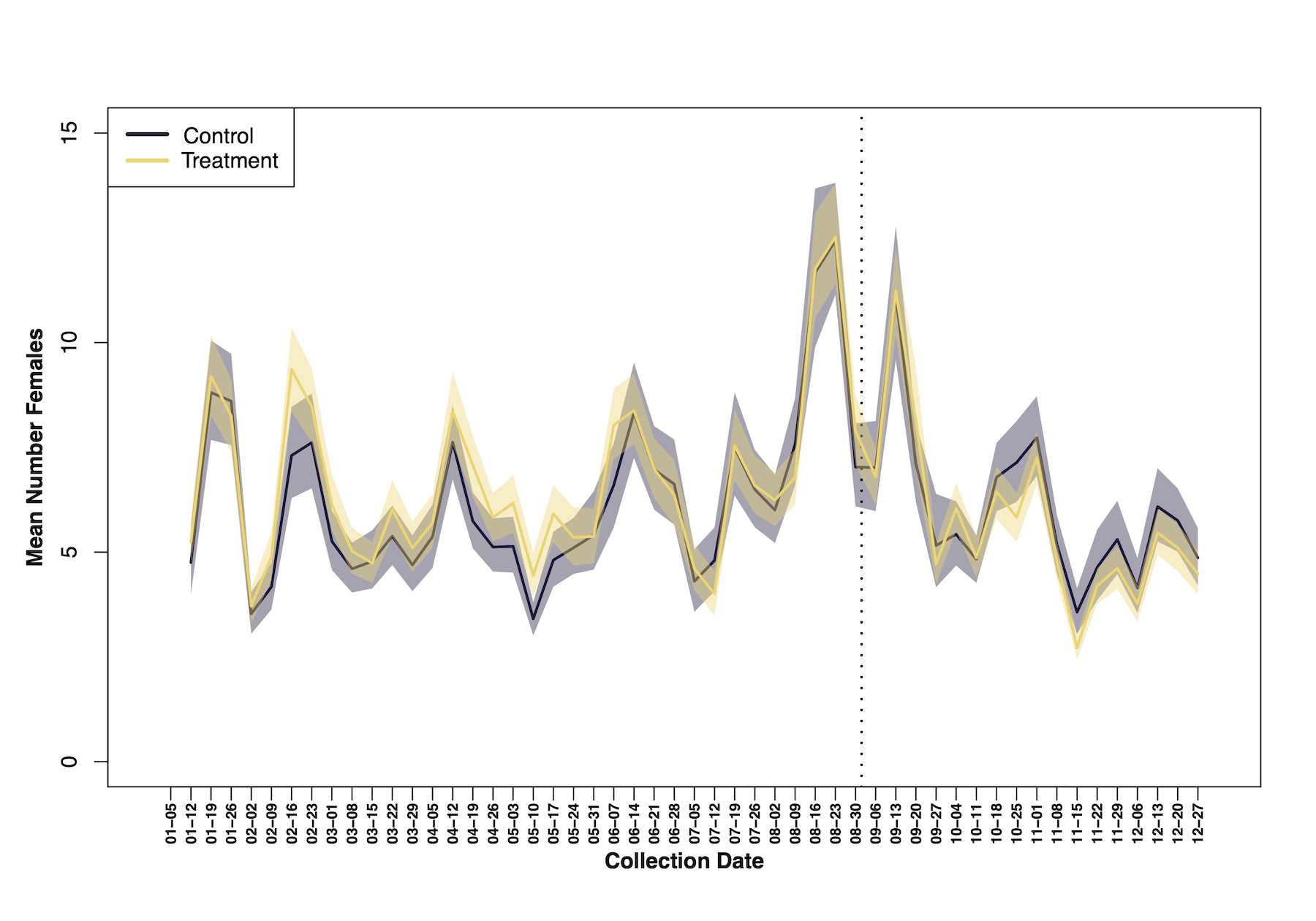

Supplement: S4 Fig — Shaded area indicates 95% bootstrap confidence interval. Dotted line shows when releases began in treatment clusters. (TIF) [file pntd.0012839.s004.tif]

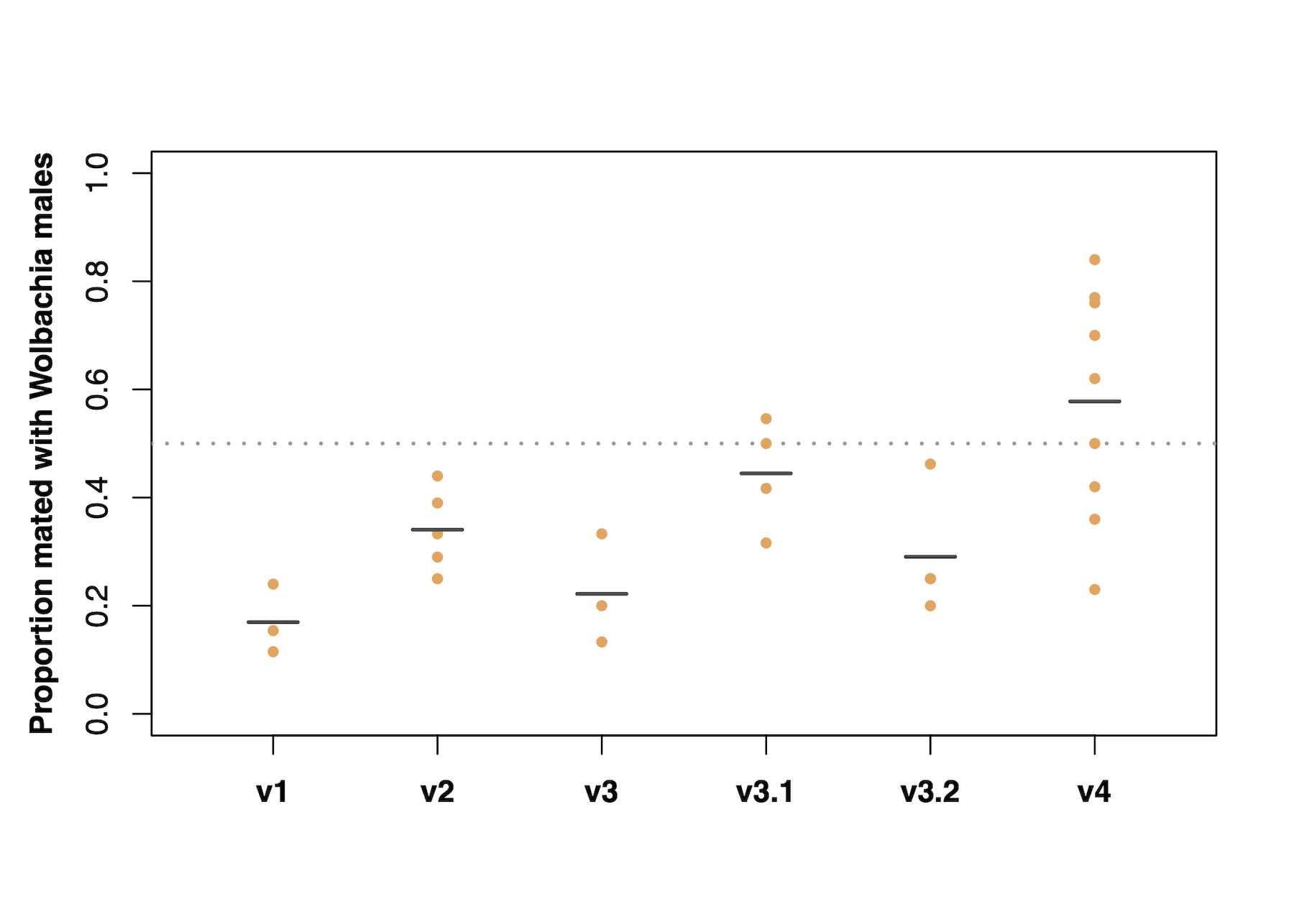

Supplement: S5 Fig — For each assay, shipping was simulated by first packing Ae. aegypti wAlbB Wolbachia males under the normal protocol, but then placing the males inside the prototype shipping container. Shipping duration ranged between 20 and 24 hours. (TIF) [file pntd.0012839.s005.tif]
